# A QIIME2-based workflow for multi-amplicon 16S rRNA profiling

**DOI:** 10.1128/mra.00836-25

**Published:** 2025-12-09

**Authors:** Armando G. Licata, Marica Zoppi, Chiara Dossena, Federico Rossignoli, Davide Rizzo, Luca Bergamaschi, Olga Nigro, Stefano Chiaravalli, Maura Massimino, Loris De Cecco

**Affiliations:** 1 Integrated Biology of Rare Tumors, Department of Experimental Oncology, Fondazione IRCCS Istituto Nazionale dei Tumori 9329, Milan, Italy; 2 Pediatric Oncology Unit, Fondazione IRCCS Istituto Nazionale dei Tumori 9329, Milan, Italy; Loyola University Chicago, Chicago, Illinois, USA

**Keywords:** 16S RNA, QIIME, bioinformatics, multi‑amplicon sequencing, stool microbiome, pediatric tumors, open‑source software

## Abstract

We present an open-source QIIME2 pipeline for 16S multi-amplicon sequencing. Benchmarked against proprietary software with a mock community, our workflow demonstrates comparable sequencing depth and taxonomic accuracy (F1-Score=0.875). The multi-region approach outperforms single amplicons, validating our pipeline as a robust alternative for semiconductor-based sequencing data.

## ANNOUNCEMENT

Multi-amplicon sequencing enhances taxonomic resolution, but robust, open-source workflows for Ion Torrent data are not well-benchmarked against proprietary tools. To address this, we present and validate a QIIME2-based (1) pipeline against the Ion Reporter (IR) software using the ZymoBIOMICS Microbial Community DNA Standard (Zymo Research, cat.no: D6306).

Amplicon libraries targeting six 16S rRNA hypervariable regions (V2;V3;V4;V6-7;V8;V9) were generated from the mock DNA standard, normalized to 20 ng/µL, using the Ion 16S Metagenomics Kit (Thermo Fisher) with both primer pools per the manufacturer’s protocol (Pub.No:MAN0010799). Libraries were indexed using Ion Xpress Adapters (Thermo Fisher), pooled, and sequenced on an Ion GeneStudio S5 platform, generating (170–350 bp) single-end reads.

Raw reads were analyzed in parallel using the proprietary IR (v5.20) Metagenomics 16S v1.1 workflow and our custom QIIME2 (v2023.7) pipeline. Raw data were demultiplexed using the Ion Torrent Suite and subsequently deconvolved into per-region FASTQs with the Metagenomics PP plugin (Thermo Fisher), which trims both barcodes and primers. Read quality was visualized to determine region-specific truncation lengths (170–210 bp), and denoising was performed with DADA2 (*2) with “-pyro*” option to correctly model Ion Torrent error profiles. The resulting region-specific amplicon sequence variant (ASV) tables were merged, summing overlapping read counts, along with their corresponding representative sequences. A phylogenetic tree was constructed with SEPP (3). For our primary benchmark, taxonomic classification was performed against the Greengenes v13_5 database (4). The reference database was prepared by extracting near-full-length sequences for the multi-region analysis and specific amplicons for single-region analyzes, using primers detailed in our original study (5). A “local-to-global” alignment strategy was then employed using VSEARCH (6), which globally aligns each short ASV to find its best-matching segment within the long reference sequences. Final QIIME2 artifacts were imported into R (v4.2.2) using the QIIME2 R package (v0.99.6) to create a phyloseq object (v1.46.0) (7), which was then collapsed to the genus level with tax_glom (NArm = TRUE). All subsequent statistical analyzes were performed in R.

To validate the performance of our pipeline, we conducted a direct comparison against the proprietary IR workflow using a mock community with a known composition. The raw data set was first characterized using FastQC (v0.12.1), revealing a total of 180,253 reads across all samples and a read length range of 50–350 bp. With the pipeline’s output established, we then assessed its taxonomic accuracy against the known mock composition (Table 1). Our V2-9 pipeline performed identically to IR, achieving a final F1-Score of 0.875. The failure of both pipelines to detect *Escherichia* is a known Greengenes v13_5 database limitation, an issue we address in our original work (5).

**TABLE 1:** Comparative performance QIIME2 and IR pipelines on a mock community^*a*^

Pipeline	Region	Number of ASVs	True positive	False positive	False negative	Sensitivity	Precision	F1 score
IR	IR	N/A^*b*^	7	1	1	0.875	0.875	0.875
QIIME	V2-9	65	7	1	1	0.875	0.875	0.875
QIIME	V2	21	7	0	1	0.875	1	0.933
QIIME	V3	14	5	0	3	0.625	1	0.769
QIIME	V4	8	6	0	2	0.75	1	0.857
QIIME	V6-7	10	5	0	3	0.625	1	0.769
QIIME	V8	16	5	0	3	0.625	1	0.769
QIIME	V9	3	1	0	7	0.125	1	0.222

^*a*^ Metrics were calculated at the genus level against the known composition of the ZymoBIOMICS mock community. True Positive (TP): genera correctly identified; False Positive (FP): genera incorrectly identified; False Negative (FN): expected genera that were missed. Sensitivity = TP/(TP+FN); Precision = TP/(TP+FP). The F1-Score is the harmonic mean of Sensitivity and Precision.

^*b*^ N/A, not available.

**Fig 1:**
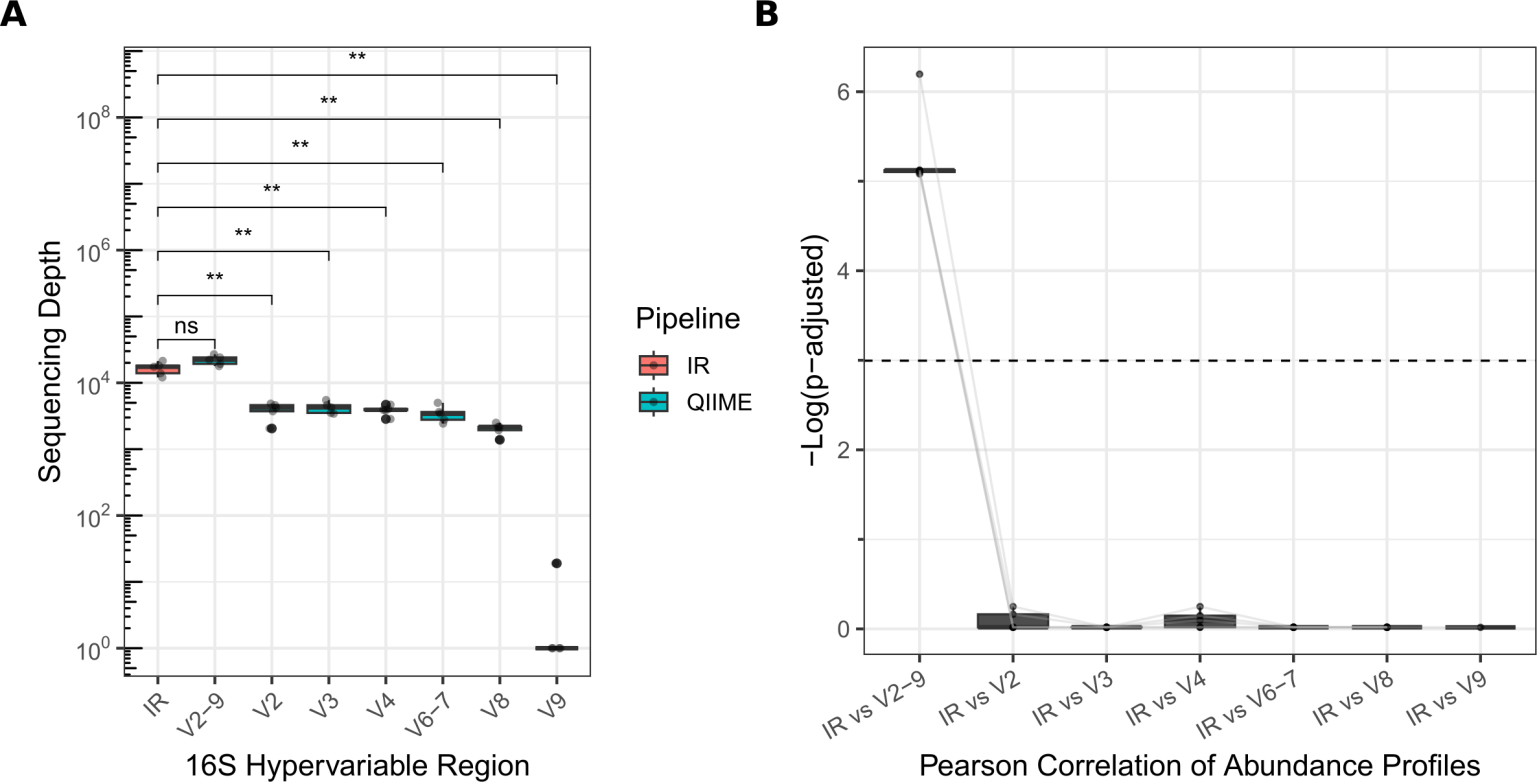
Technical Validation of the QIIME2 Pipeline Against IR. (**A**) Boxplots show the distribution of sequencing depth per sample for the IR pipeline and for each QIIME2 configuration. Statistical significance was determined by a Wilcoxon rank-sum test. (**B**) Results of pairwise Pearson correlations performed on genus-level relative abundance profiles. Relative abundances were calculated by converting raw counts to proportions for each sample. The y-axis displays the -Log₁₀ transformed, BH-adjusted p-values; higher values indicate a stronger, more significant correlation. The dashed line represents a significance threshold of *P* = 0.005. Significance is denoted as: ns, *P* > 0.05; **, *P* ≤ 0.01.

Our merged multi-region (V2-9) profile achieved a median sequencing depth comparable to the IR pipeline (Fig. 1A). Furthermore, the V2-9 genus-level relative abundance profile was highly and significantly correlated with the IR profile, indicating strong methodological agreement (Fig. 1B). This high concordance was unique to the merged profile, as all single-region analyzes showed weaker performance and were not significantly correlated. These results validate our QIIME2 pipeline as a comparable and accurate alternative for multi-amplicon sequencing.

## Data Availability

The complete QIIME2 pipeline for Ion Torrent data analysis—including single- and multi-region workflows—is available at: https://github.com/DeCeccoLAB/QIIME2-MultiRegion-Microbiome-Pipeline. This repository also hosts a fully dockerized QIIME2 v2023.7 environment, detailed documentation of all bioinformatic steps, parameters, and scripts, as well as the processed datasetdata set used in this study. All raw 16S rRNA sequencing data (region-deconvoluted) are publicly available in the NCBI GEO under accession number (GSE300047).

## References

[1] Bolyen E, Rideout JR, Dillon MR, Bokulich NA, Abnet CC, Al-Ghalith GA, Alexander H, Alm EJ, Arumugam M, Asnicar F, et al.. 2019. Reproducible, interactive, scalable and extensible microbiome data science using QIIME 2. Nat Biotechnol 37:852–857. doi:10.1038/s41587-019-0209-9 31341288 PMC7015180

[2] Callahan BJ, McMurdie PJ, Rosen MJ, Han AW, Johnson AJA, Holmes SP. 2016. DADA2: high-resolution sample inference from Illumina amplicon data. Nat Methods 13:581–583. doi:10.1038/nmeth.3869 27214047 PMC4927377

[3] Mirarab S, Nguyen N, Warnow T. 2011. SEPP: SATé-Enabled Phylogenetic Placement. Proceedings of the Pacific Symposium; Kohala Coast, Hawaii, USA: , p 247–258 World Scientific. doi:10.1142/9789814366496_0024 22174280

[4] DeSantis TZ, Hugenholtz P, Larsen N, Rojas M, Brodie EL, Keller K, Huber T, Dalevi D, Hu P, Andersen GL. 2006. Greengenes, a chimera-checked 16S rRNA gene database and workbench compatible with ARB. Appl Environ Microbiol 72:5069–5072. doi:10.1128/AEM.03006-05 16820507 PMC1489311

[5] Licata AG, Zoppi M, Dossena C, Rossignoli F, Rizzo D, Marra M, Gargari G, Mantegazza G, Guglielmetti S, Bergamaschi L, Nigro O, Chiaravalli S, Massimino M, De Cecco L. 2025. QIIME2 enhances multi-amplicon sequencing data analysis: a standardized and validated open-source pipeline for comprehensive 16S rRNA gene profiling. Microbiol Spectr 13:e0167325. doi:10.1128/spectrum.01673-25 40711419 PMC12403817

[6] Rognes T, Flouri T, Nichols B, Quince C, Mahé F. 2016. VSEARCH: a versatile open source tool for metagenomics. PeerJ 4:e2584. doi:10.7717/peerj.2584 27781170 PMC5075697

[7] McMurdie PJ, Holmes S. 2013. Phyloseq: an R package for reproducible interactive analysis and graphics of microbiome census data. PLoS One 8:e61217. doi:10.1371/journal.pone.0061217 23630581 PMC3632530

